# Supplementary material for: A Global Screen for Assembly State Changes of the Mitotic Proteome by SEC-SWATH-MS
Source: Cell Syst. 2020 Feb 26;10(2):133–155.e6. doi: 10.1016/j.cels.2020.01.001 (PMC7042714; doi:10.1016/j.cels.2020.01.001)

O95562 | SFT2B_HUMAN | SFT2D2 UNQ512/PRO1027
Monomer MW [kDa]: 17.779 Monomer expected elution fraction: 55

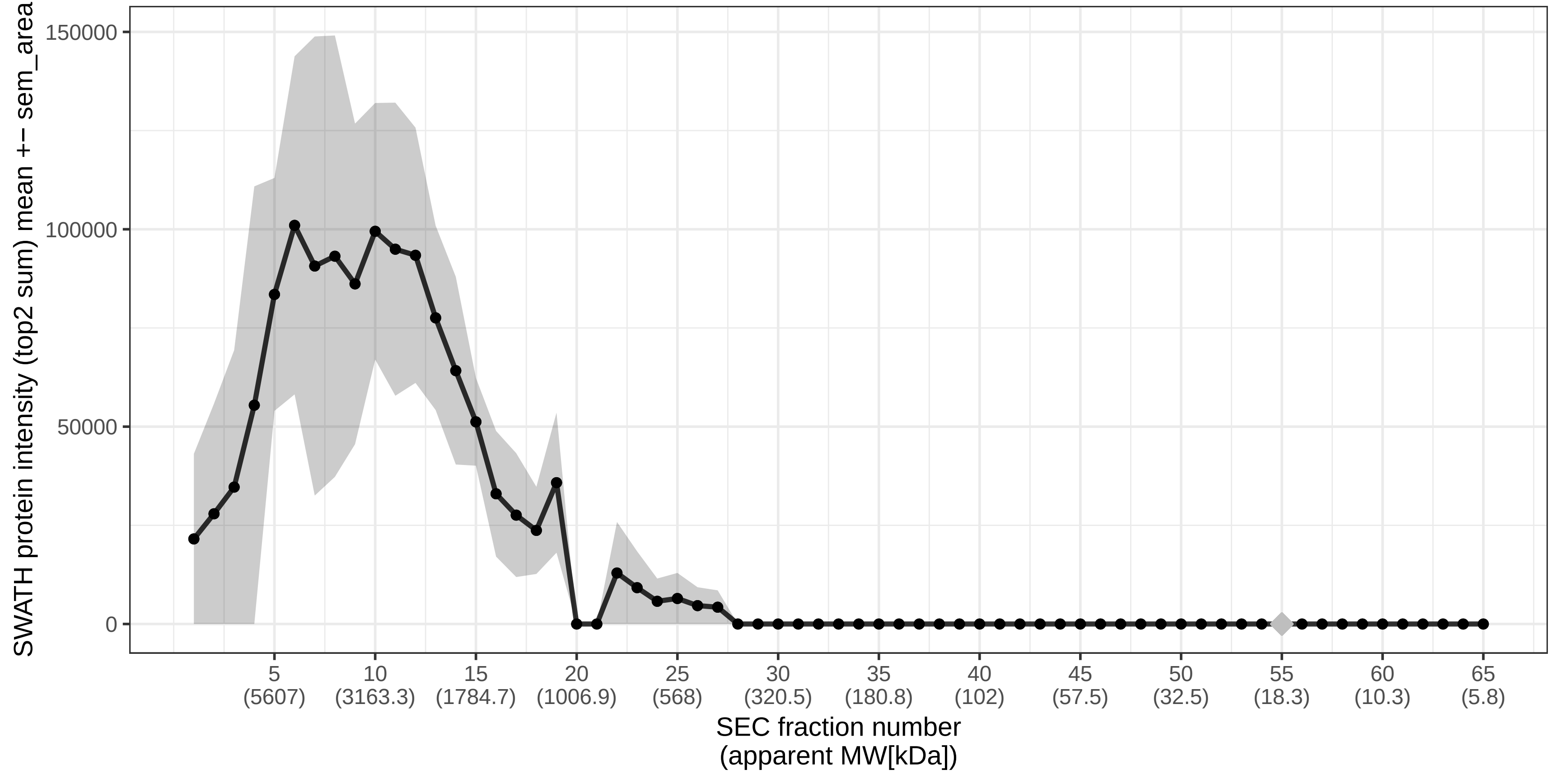

Supplement: Data S1. SEC-SWATH-MS Protein Chromatograms, Related to Figure 1 [file mmc6.zip › SECchrom_O95562_SFT2B_HUMAN_SFT2D2_UNQ512_PRO1027.pdf]
